# A case of late-onset Dowling-Degos disease with hidradenitis suppurativa

**DOI:** 10.1016/j.jdcr.2025.08.026

**Published:** 2025-09-05

**Authors:** Catherine F. Sollitto, Claire Wolinsky

**Affiliations:** a New York Institute of Technology College of Osteopathic Medicine, Old Westbury, New York; b Department of Dermatology, Mount Sinai Hospital, New York, New York

**Keywords:** breast cancer, chemotherapy, Dowling-Degos disease, hidradenitis suppurativa, NOTCH mutation

## Introduction

Dowling-Degos disease (DDD) is a rare autosomal dominant genodermatosis that typically presents in puberty or early adulthood, most commonly during the third to fourth decades of life. Clinically, it is characterized by progressive brown-to-black hyperpigmented macules and papules in flexural sites, primarily the axillae, groin and inframammary folds. Here, we report an unusual case of sporadic concomitant hidradenitis suppurativa (HS) and DDD in the axillary, inguinal, and vulvar regions arising in the patient’s 6th decade of life after having received radiation and chemotherapy.

## Case report

**Fig 1:**
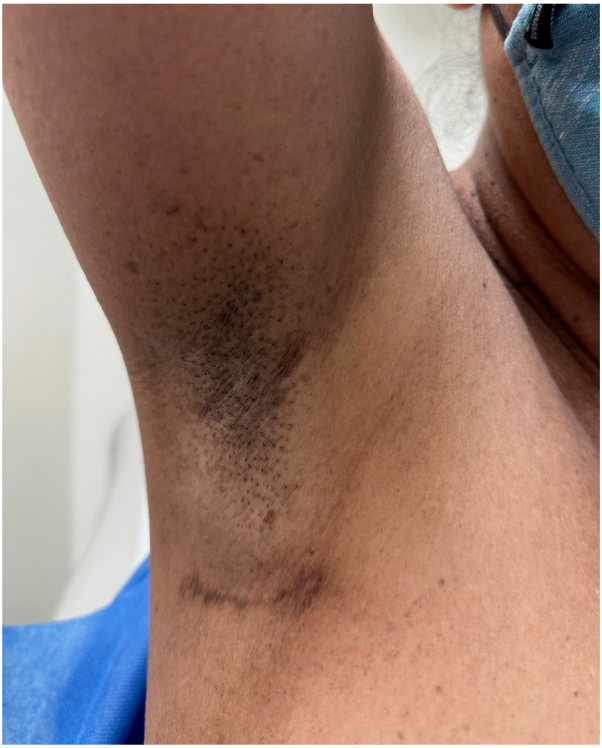
Macular hyperpigmentation and scarring in the axilla.

**Fig 2:**
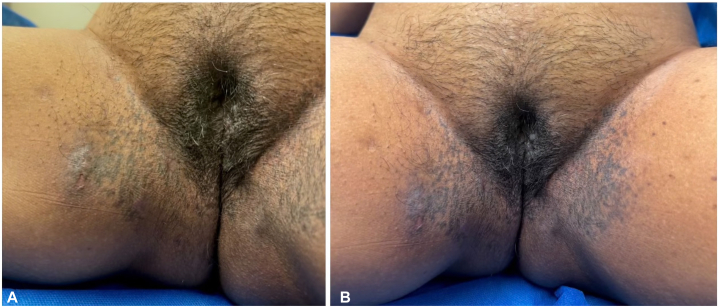
**A** and **B,** Macular reticulated hyperpigmentation of the inner thighs and vulva with scarring and HS ulcers.

A 54-year-old woman of South Asian descent with a history of HS presented to the dermatology clinic with pigmented fistulae and coalescing hyperpigmented macules in the axillary region (Fig 1) accompanied by macular reticulate hyperpigmentation and ulcers in the inguinal and vulvar regions (Fig 2, *A* and *B*). She reported onset of both the ulcerative lesions and pigmentation around the age of 50 following multiple chemotherapy and radiation treatments for a right breast carcinoma. The patient reported significant discomfort from the HS lesions but no pain or distress from the surrounding hyperpigmentation. The patient reported no family history of HS or DDD and denied history of nicotine or tobacco use. Her medications included daily use of oral doxycycline (100 mg BID) and clindamycin lotion to control her HS. Upon clinical examination, the patient had fistulae and hyperpigmented macules coalescing into patches in the axillae as well as symmetrical, reticulated macular hyperpigmentation surrounding HS ulcers within the vulvar and inguinal regions (Figs 1 and 2, *A* and *B*).

A shave biopsy of the right inner thigh showed epidermal hyperplasia accompanied by dilated follicular infundibula, hyperpigmentation of basal keratinocytes and increased melanophages. Based on the histological and clinical findings, a diagnosis of DDD was made in association with Hurley Stage II HS.

## Discussion

Despite its rarity, reports of DDD with simultaneous HS have been documented as early as 1990.^1^ Since then, various other presentations have been published with the majority describing classic patterns of inheritance and distribution.^2^ Unlike the classic presentation of HS and DDD, our patient reports unusually late disease onset, with symptoms emerging during the 6th decade of life, as well as involvement of the vulvar region which is seldom mentioned in the literature. Notably, our patient also denied any family history of either condition.

Although HS and DDD have been previously reported to coexist, there are very few cases documenting the coexistence of both in a patient who lacks a family history of either condition.^2^, ^3^, ^4^ Such cases reported by Nokdhes et al and Del Mar et al both document typical disease timelines with onset prior to the fouth decade and do not specify genital involvement of HS and DDD together. Moreover, specific mention of simultaneous HS and DDD affecting the vulva is scarce within the literature; Dupont et al is among one of the only cases, which explicitly states vulvar involvement and is supported by accompanying figures.^3^ Other publications, such as that by Agut-Busquet et al, have alluded to genital involvement but are less clear in describing exact distribution. In their 2019 study, they documented “gluteal and pubic involvement” in 53.33% of patients with HS + DDD along with figures that had illustrated the perineal area being affected; yet, it is uncertain if the genitalia was also included in such patients.^5^ There are, however, various other reports that have been made regarding the presentation of DDD on the vulva but fail to mention the presence of concomitant HS.^6^, ^7^, ^8^

The presentation of HS and DDD in the same patient substantiates previous research regarding the related pathogenesis of the 2 conditions. Deficient NOTCH pathway signaling has been implicated in both HS and DDD, resulting in impaired epidermal and follicular hyperkeratosis as well as abnormal melanocyte and keratinocyte morphology. Specifically, mutations in PSENEN, POFUT1, and NCSTN are most often linked to cases of DDD in association with HS.^9^

The clinical timeline suggests that the patient’s cancer treatment may have played a contributory role in HS/DDD onset. Certain cancers such as squamous cell carcinoma (SCC) are known to harbor NOTCH-inactivating mutations, which may contribute to the elevated risk of SCC observed in patients with HS.^10^ While mutations in both NOTCH1 and NOTCH2 expression have been associated with increased breast cancer incidence and poorer prognosis, the targeted effects of chemotherapeutic agents on NOTCH signaling lack sufficient investigation.^11^ A comprehensive PubMed search using terms “NOTCH” or “PSENEN” or “POFUT1” or “NCSTN” with “chemotherapy” and “radiation therapy” did not yield any pertinent results directly linking these treatment modalities to suppressed pathway signaling or HS/DDD development.

It should be noted that the lack of genetic testing in our patient is a limitation to this study, especially considering the spontaneous presentation and cancer treatment timeline. Further studies would also be required to determine whether patients with HS and/or DDD may be at increased risk for developing other cancers such as breast cancer, or if targeted anti-cancer therapies induce genomic alterations that put patients at risk for later developing sporadic HS/DDD.

## Conflicts of interest

None disclosed.
